# Conformational changes in and translocation of small proteins: insights into the ejection mechanism of podophages

**DOI:** 10.1128/jvi.01249-24

**Published:** 2024-12-20

**Authors:** Jing Zheng, Hao Xiao, Hao Pang, Li Wang, Jingdong Song, Wenyuan Chen, Lingpeng Cheng, Hongrong Liu

**Affiliations:** 1 Institute of Interdisciplinary Studies, Key Laboratory for Matter Microstructure and Function of Hunan Province, Key Laboratory of Low-dimensional Quantum Structures and Quantum Control, School of Physics and Electronics, Hunan Normal University 12568 , Changsha, China; 2 National Key Laboratory of Intelligent Tracking and Forecasting for Infectious Diseases, National Institute for Viral Disease Control and Prevention, Chinese Center for Disease Control and Prevention 12415 , Beijing, China; 3 Department of Microbiology, College of Life Sciences, Hunan Normal University 554899 , Changsha, China; Michigan State University, East Lansing, Michigan, USA

**Keywords:** bacteriophages, core proteins, trans-envelope channel, dsDNA ejection

## Abstract

Podophage tails are too short to span the cell envelope during infection. Consequently, podophages initially eject the core proteins within the head for the formation of an elongated trans-envelope channel for DNA ejection. Although the core proteins of bacteriophage T7 have been resolved at near-atomic resolution, the mechanisms of core proteins and DNA ejection remain to be fully elucidated. In this study, we provided improved structures of core proteins in mature T7 and the portal–tail complex in lipopolysaccharide-induced DNA-ejected T7 to resolutions of approximately 3 Å. Using these structures, we identified three small proteins, namely gp14, gp6.7, and gp7.3, and illustrated the conformational changes in and translocation of these proteins from the mature to DNA-ejected states. Our structures indicate that gp6.7, which participates in the assembly of the core and trans-envelope channel, is a core protein, and that gp7.3 serves as a structural scaffold to assist the assembly of the nozzle into the adaptor.

**IMPORTANCE:**

Podophage T7 core proteins form an elongated trans-envelope channel for genomic DNA delivery into the host cell. The structures of the core proteins within the mature T7 and assembled in the periplasmic tunnel form in the DNA-ejected T7 have been resolved previously. Here, we resolved the structures of two new structural proteins (gp6.7 and gp7.3) within mature T7 and receptor-induced DNA-ejected T7. The gp6.7 protein participates in the assembly of the core complex within mature T7 and the trans-envelope channel during T7 infection; therefore, gp6.7 is a core protein. Before T7 infection, gp7.3 plays a role in promoting the assembly of the nozzle into the adaptor.

## INTRODUCTION

Many double-stranded DNA (dsDNA) viruses, including tailed bacteriophages, herpesviruses, and adenoviruses, have a dodecameric portal attached to a unique pentameric vertex of their icosahedral capsid shell for genomic DNA package and delivery (1–3). In tailed bacteriophages, the tail is attached to the isometric icosahedral or elongated icosahedral head through the portal. During viral DNA packaging, tailed bacteriophages encapsidate DNA in the head through the portal channel. In mature phages, the portal connects to a tail, which retains DNA within the capsid, recognizes host cell receptors, and delivers the genome into the cytoplasm during infection (4). In most tailed bacteriophages, the last packaged dsDNA end, which is located in the portal–tail channel of the mature phage virion, is the first to be ejected during genome delivery (5). Compared with myophage and siphophage tails (6–8), Gram-negative podophage tails (9), which typically consist of a hexameric nozzle, one to three dodecameric adaptors, and one to three types of fibers (10, 11), are too short to span the cell envelope for DNA ejection during infection. Consequently, Gram-negative podophages usually employ a special strategy for DNA ejection in which a number of ejection proteins (also known as core proteins or pilot proteins) within their head are ejected first, forming an elongated channel across the cell envelope for DNA ejection (12–16). However, the mechanisms of core proteins and DNA ejection remain to be fully elucidated due to limited structural and biophysical information on core proteins in podophages (10, 13, 17).

The bacteriophage T7, *Escherichia coli* podophage, has been employed as a model for the investigation of the DNA packaging and delivery mechanism among tailed phages and related dsDNA viruses (16, 18–23). T7 has an incomplete T = 7 icosahedral capsid shell, comprising 415 copies of the capsid shell protein gp10. The tail, connected to a unique vertex of the head by the dodecameric portal gp8, consists of the dodecameric adaptor protein gp11 and the hexameric nozzle protein gp12 (20, 24) surrounded by six trimeric tail fibers gp17 (16, 22, 24). An internal cylinder-shaped core complex is attached to the top of the portal within the head. The core is formed by the core proteins gp14, gp15, and gp16; upon virion adsorption onto host membranes or the receptor rough lipopolysaccharide (LPS) (25), these core proteins have been suggested to extend the tail channel and form a trans-envelope channel for genome delivery into the infected cell (26–29). Among Gram-negative podophages, gp14 is the most conserved of the three core proteins (17). We previously reported the structures of mature and DNA-ejected T7 at near-atomic and subnanometer resolutions, respectively (23). The core in mature T7 is formed by a four-fold gp16 ring, an eight-fold gp15 ring, and a less-well-defined eight-fold spoke, which could be formed by gp14 (23). In the DNA-ejected T7, core proteins are ejected from the head through the portal–tail channel forming an extended channel, in which gp14 is embedded in the nozzle and span the outer membrane (23), gp15 is ejected into the periplasm, and gp16 extends into the host cytoplasm (23, 30, 31). Biochemical data has demonstrated that upon the initiation of infection, two small proteins, gp6.7 and gp7.3, identified inside the head and the tail channel, respectively, were also ejected from the T7 virion along with the three core proteins (27). However, the structures and precise locations of the two small proteins remained unknown. Whether gp6.7 forms a part of the internal core is unclear, and the roles of the two small proteins in DNA delivery remain to be characterized.

In this study, using a 300 kV cryo-electron microscopy (cryo-EM) and defocus refinement with Z-height correction, we provided improved structures of core proteins at a resolution of 3 Å, the portal–tail complex in mature T7 at a resolution of 2.7 Å, and the portal–tail complex in LPS-induced DNA-ejected T7 at a resolution of 3.5 Å. We built atomic models for gp14, gp6.7, and gp7.3 and illustrated the translocation of and conformational changes in these proteins from the mature to DNA-ejected states. Our structures suggest that gp6.7 is a core protein and that gp7.3 as a structural scaffold promotes the assembly of the nozzle into the adaptor.

## RESULTS

### Overall structures of mature and DNA-ejected T7

**Fig 1:**
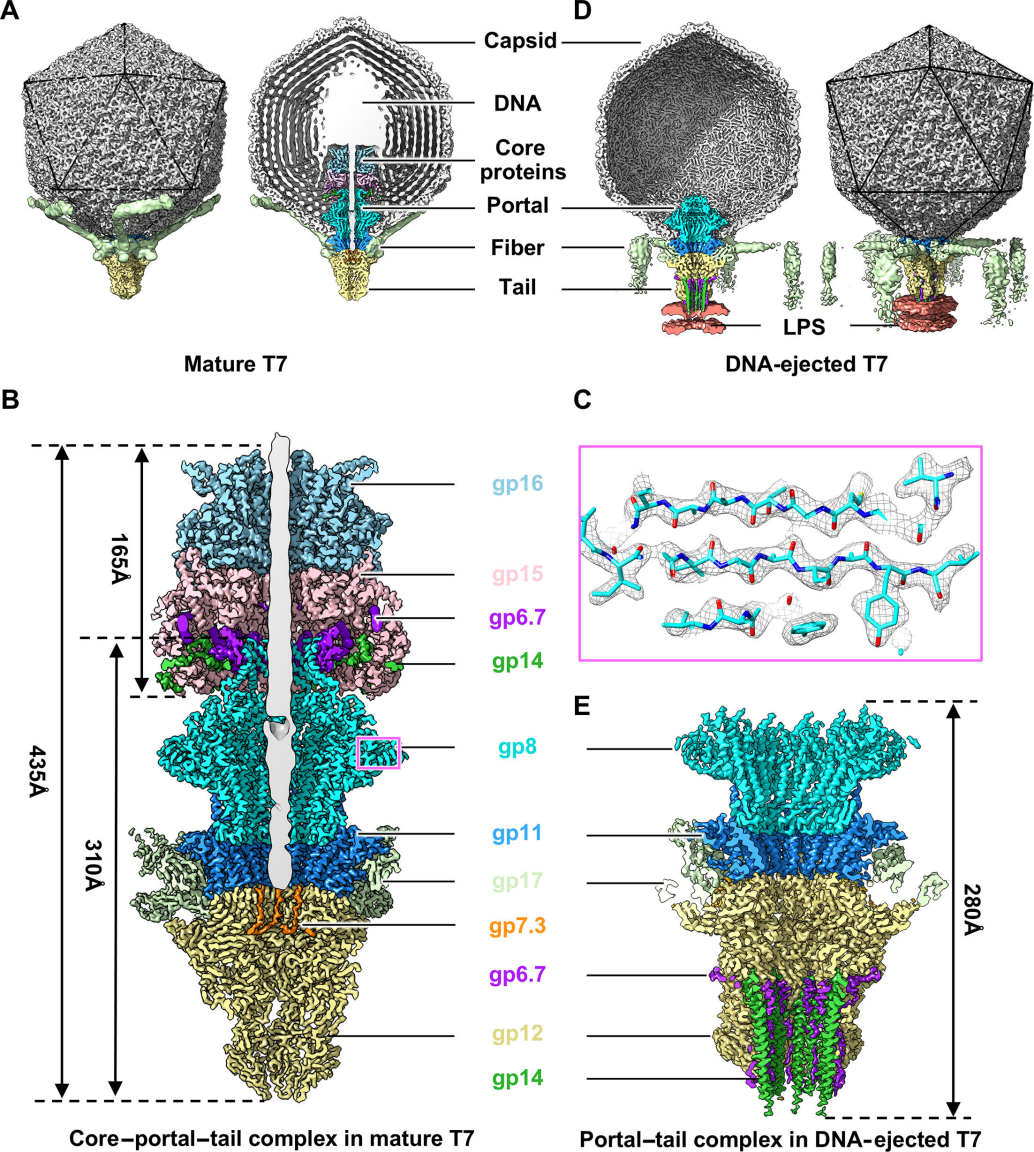
Overall structures of mature and DNA-ejected T7. (**A**) Side (left) and cut-open (right) views of the entire asymmetric structure of mature T7. Color code applies to panels A–E. (**B**) Cut-open view of high-resolution density map of the core–portal–tail complex in mature T7. (**C**) Superposition of our atomic models (sticks) on density maps (mesh) extracted from box (portal) in panel B. (**D**) Side (left) and cut-open (right) views of the entire asymmetric structure of DNA-ejected T7. (**E**) Cut-open view of high-resolution density map of the portal–tail complex in DNA-ejected T7.

For the collection of cryo-EM data, mature T7 particles were purified from *E. coli*. The DNA-ejected T7 particles were then obtained by incubating the purified mature T7 with rough LPS. A total of 75,594 mature particles and 23,461 DNA-ejected particles were extracted from 2,221 and 4,401 cryo-EM micrographs, respectively (Fig. S1A and B). Using the symmetry–mismatch reconstruction method (24, 32), we obtained an asymmetric structure of the entire mature T7 particle at a resolution of 6.1 Å (Fig. 1A; Fig. S1C). The mature T7 contains an icosahedral head encapsulating dense dsDNA, a cylinder-shaped core protein complex, dodecamers of both portal and adaptor, and a hexameric tail nozzle flanked by six trimeric tail fibers (Fig. 1A). Through the local reconstruction method (23, 24) and by independently refining the Z-height for different regions, we obtained the structures of the core complex, portal, and portal–tail complex in mature T7 at resolutions of 3, 2.5, and 2.7 Å, respectively (Fig. 1B and C; Fig. S1D). The resolutions were estimated using the Fourier shell correlation = 0.143 criterion according to the gold standard method (33). Data acquisition and reconstruction statistics are shown in Table S1. Compared with our previous structures of mature T7 (23), the resolutions of core proteins in this study were remarkably improved (Fig. S2). Notably, we identified eight copies of the core protein gp14 and 12 copies of the small protein gp6.7 between the portal barrel and the core, as well as six copies of the small protein gp7.3 at the junction of the adaptor and tail nozzle within the tail channel, which served to anchor the end of the dsDNA (Fig. 1B). The atomic models of the portal–tail complex in our latest study are identical to those of our previous study on T7 (Fig. S3 and S4) (23).

We also reconstructed an asymmetric structure of the DNA-ejected T7 complexed with LPS at a resolution of 8.2 Å and the local structure of the portal–tail complex at a resolution of 3.5 Å (Fig. 1D and E; Fig. S1C and D). The structure of the entire DNA-ejected T7 comprises an empty icosahedral head, a portal–tail complex, and an extended tail channel that interacts with LPS (Fig. 1D). Twelve copies of gp6.7 with six copies of the core protein gp14 form a tight and extended channel located symmetrically within the tail nozzle and interacting with LPS (Fig. 1D and E; Fig. S5).

### Structure of the core in mature T7

**Fig 2:**
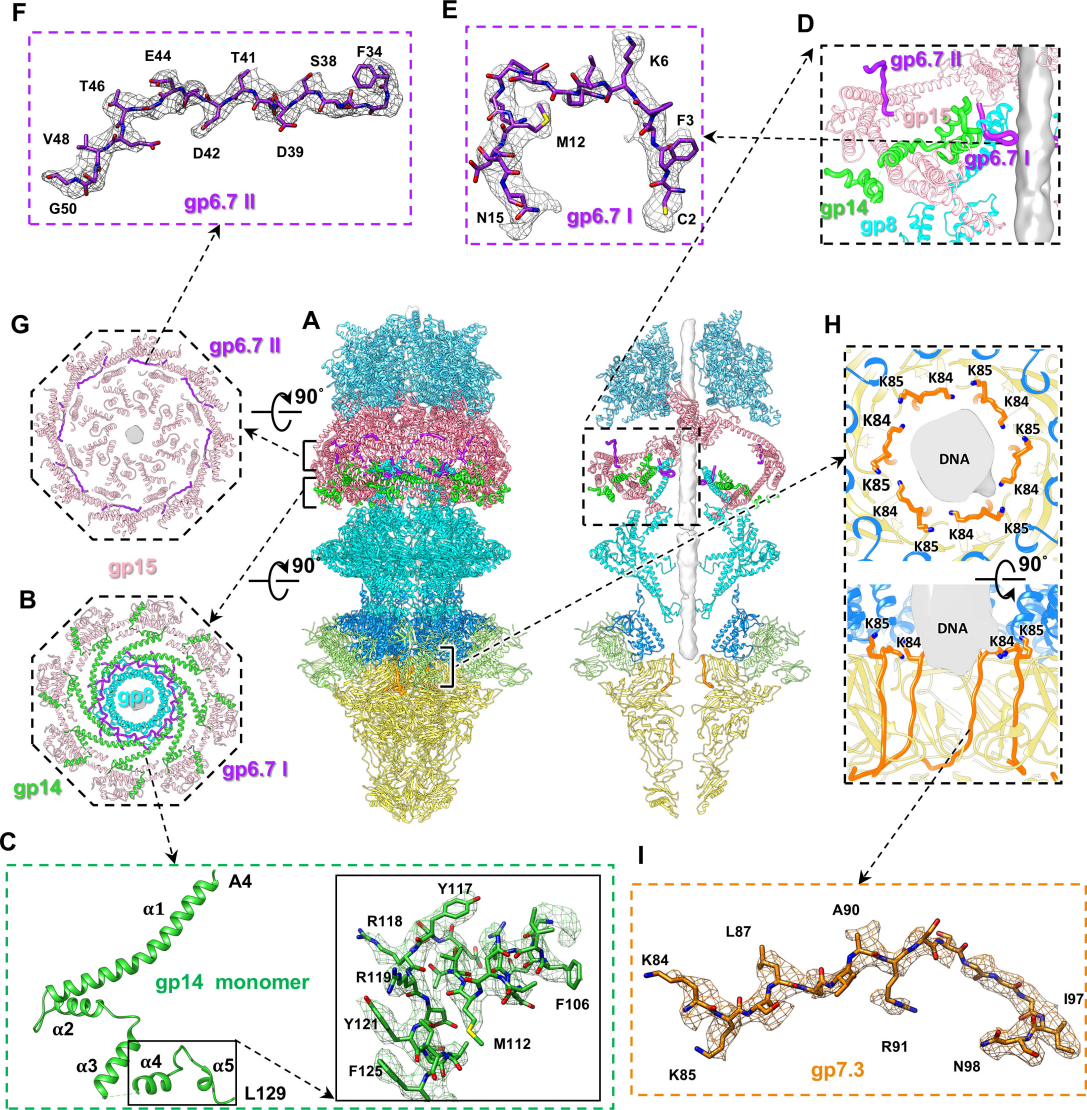
Structure of core–portal–tail complex in mature T7. (**A**) Side (left) and slab (right) views of atomic models (ribbons) of core–portal–tail complex in mature T7. Color coding is identical to that in Fig. 1B. Middle gray rod is a density map of DNA. (**B**) Cross-section view of columns in A showing interactions among portal and proteins gp6.7, gp14, and gp15. (**C**) Ribbon model (left) of gp14 molecule and zoomed-in view (right) of atomic model superimposed on its density map (mesh). (**D**) Zoomed-in view of interactions among proteins gp6.7, gp8, gp14, and gp15. (**E, F**) Atomic models (sticks) of gp6.7 loop I (residues 2–15, **E**) and loop II (residues 34–50, **F**) superimposed on their density maps (mesh). (**G**) Cross-section view of columns in A showing interactions between gp6.7 and gp15. (**H**) Top (upper) and cut-open (bottom) views of interactions between dsDNA and protein 7.3. Residues K84 and K85 of gp7.3 are labeled. (**I**) Atomic model (stick) of gp7.3 superimposed on its density map (mesh).

The structures of the eight-fold gp15 ring and a four-fold gp16 ring are identical to our previously resolved structures (23). These rings form an α-helix-rich tapered cylinder-shaped structure, resembling an upside-down cup attached to the portal helical barrel (Fig. 2A). On the basis of our improved density map, we identified eight copies of gp14 between the gp15 ring and the portal helical barrel (Fig. 2A and B). We also modeled 112 amino acid residues (4–86 and 101–129) of the 196-residue gp14 protein (Fig. 2C). Each gp14 molecule is composed of five α-helices connected by four loops (Fig. 2C). The longest helix α1 and a shorter helix α2 in the gp14 molecule enwind the portal barrel, bridging the portal barrel and the sidewall of the cylinder formed by gp15 (Fig. 2B; Fig. S6A and B). Helix α3 traverses through a hole on the sidewall, and helices α4 and α5 bind to the outer sidewall of the cylinder, which is surrounded by the genomic DNA (Fig. 1A; Fig. S6A and C). The C-terminal domain (residues 130–196) could not be resolved, probably due to its asymmetric interaction with the genomic DNA (Fig. S7).

We resolved two loops of the 88-residue gp6.7 protein: loop I (residues 2–15) and loop II (residues 34–50) (Fig. 2D through F) by using the local reconstruction focusing on the portal and loop I, and on the complex of gp14, gp15, and loop II, respectively. Twelve copies of the gp6.7 loop I, which surrounds the outer surface of the dodecameric portal helical barrel, mediate the interaction between the portal barrel and gp14 (Fig. 2B and E). However, we did not resolve the interactions between gp6.7 loop I and gp14 at near-atomic resolution using the local reconstruction method (Fig. 2D), suggesting that the interactions between the two proteins might be flexible. Intriguingly, only eight copies of the gp6.7 loop II were resolved to interact with the inner sidewall of the cylinder formed by gp15 (Fig. 2F and G). The unresolved four copies of loop II of gp6.7 are probably asymmetrically distributed around the cylinder because only eight copies of gp15 are in the cylinder. Thus, gp6.7 serves as an adapter for coordinating the symmetry mismatch between the dodecameric helical barrel domain of the portal, the eight copies of gp14, and the eight copies of gp15, thereby stabilizing the core proteins on the portal.

### Structure of gp7.3 in mature T7

We modeled the C-terminal loop (residues 84–98) of 99-residue gp7.3 (Fig. 2H and I). Six copies of the C-terminal loop, with each copy being anchored to two adjacent nozzle proteins of gp12 (Fig. S8), are located at the junction of the nozzle and adaptor in the tail channel (Fig. 2H). Twelve lysine residues (residues K84 and K85) derived from the six copies of gp7.3 interact with the dsDNA end in the portal–tail channel (Fig. 2H). In the entire asymmetric structure of mature T7 at a low threshold, some additional densities are observed within the portal–tail channel that closely interact with dsDNA (Fig. S9A and B). On the basis of the location of the C-terminus in the resolved structure of gp7.3, the unstructured densities encircling dsDNA can be attributed to the unresolved N-terminus (residues 1–83) in gp7.3 (Fig. S9B and C). The N-terminus of gp7.3 is mostly positively charged (Fig. S10). Thus, all gp7.3 molecules interact with both the tail and the DNA end within the portal–tail channel.

### Conformational changes in gp12, gp14, and gp6.7 in DNA-ejected T7

**Fig 3:**
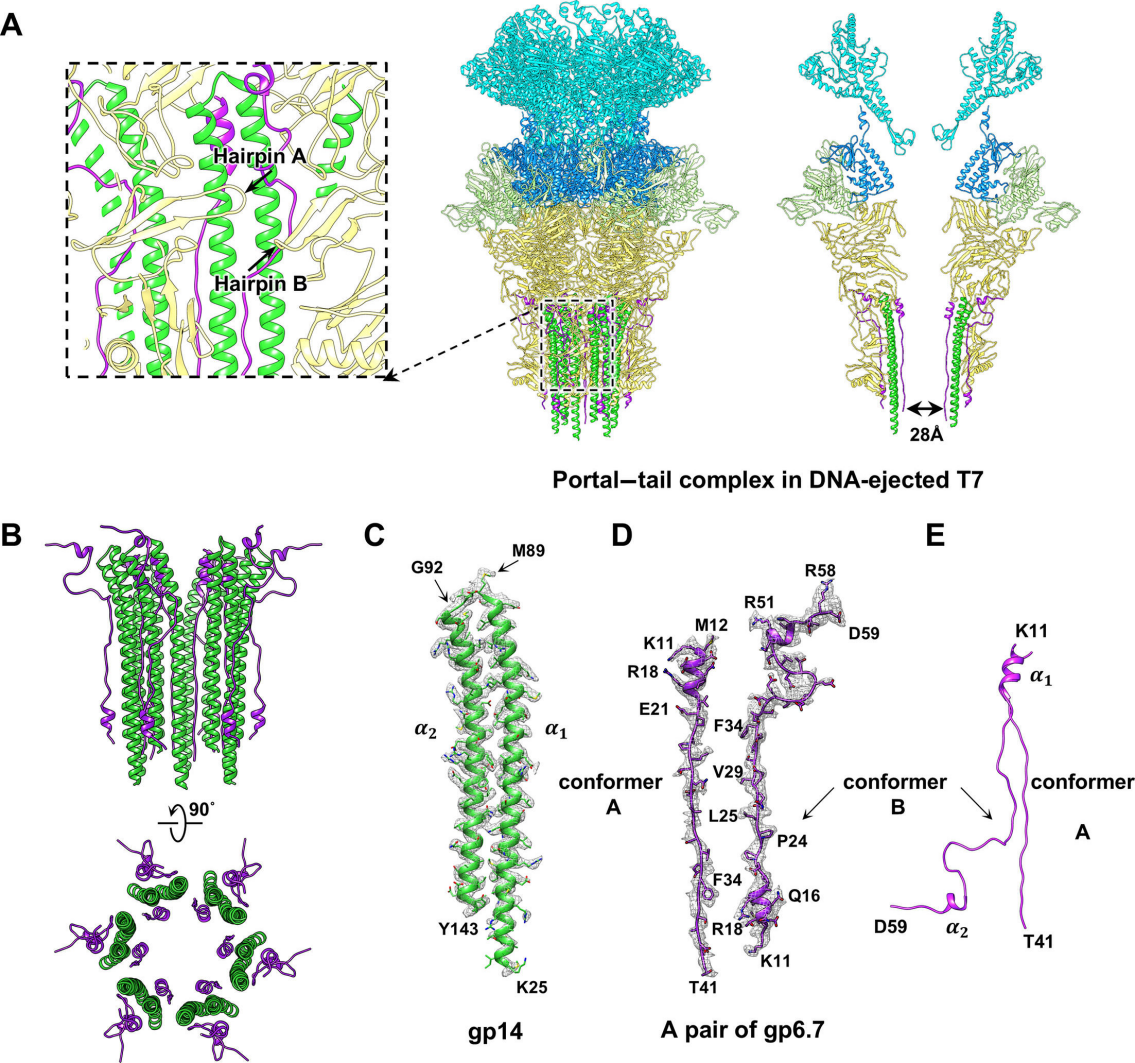
Structure of the core–portal–tail complex in DNA-ejected T7. (**A**) Side (center) and slab (right) views of atomic models (ribbons) of portal–tail complex in DNA-ejected T7. In the left inset, hairpin A from one gp12 molecule and hairpin B from adjacent gp12 molecule interact with the gp14–gp6.7 channel inside tail nozzle. Color coding is identical to that in Fig. 1D. (**B**) Side (upper) and bottom (bottom) views of atomic models (ribbon) of the gp14–gp6.7 channel. (**C**) Atomic model of gp14 superimposed on its density map (mesh). (**D**) Atomic models of conformers A and B of gp6.7 superimposed on their density maps (mesh). (**E**) Superposition of atomic models of conformers A and B of gp6.7 for showing conformational differences.

Compared with the tail structure of the mature T7, the hexameric tail nozzle of the DNA-ejected T7 undergoes conformational changes, leading to the opening of the tail channel (Fig. 1B, E, 2A and 3A; Fig. S11A). Additionally, in the tip domain of the mature T7 nozzle, each gp12 molecule contains a three-stranded β-sheet (residues 264–294) with two long hairpins (Fig. S11B): A (residues 264–282) and B (residues 283–294). Hairpin A of one gp12 molecule is close to hairpin B of the adjacent gp12 molecule (Fig. S11B). By contrast, in DNA-ejected T7, hairpin A of each gp12 molecule rotates almost 90° (Fig. S11C), leading to the formation of an extended gap (Fig. S11D) for the attachment of the gp14–gp6.7 complex (described below) in the tail channel (Fig. 3A and B).

Six copies of gp14 (residues 25–143) and twelve copies of gp6.7 (residues11-41) were resolved in the extended channel in the DNA-ejected T7 (Fig. 3A and B). Each gp14 molecule consists of two antiparallel α-helices connected by a short loop (Fig. 3C). The gp14 molecule undergoes remarkable structural change from the mature state to the DNA-ejected state (Fig. 2C and 3C; Fig. S12). In addition, only six copies of gp14 were resolved in the extended channel of the DNA-ejected T7 (Fig. 3B), in contrast to the eight copies of gp14 in the T7 mature virion (Fig. 2B).

We resolved two conformers of gp6.7 (conformer A residues 11–41, and conformer B, residues 11–59) in DNA-ejected T7 (Fig. 3D). Conformer A of gp6.7 consists of a short α-helix connected by a long loop, and conformer B consists of two short α-helices connected by a long loop (Fig. 3D). Six copies of conformer A of gp6.7 are located inside the channel. Moreover, each short α-helix of gp6.7 binds to the turn of the two antiparallel α-helices of gp14, forming a three-helix bundle, and each long loop of gp6.7 connects the cleft between two adjacent gp14 molecules (Fig. S13). Six copies of conformer B of gp6.7 are located outside the channel, staying in the opposite direction along the channel compared with the conformer A. Moreover, each short helix α1 binds to the distal end of the two antiparallel α-helices, forming a three-helix bundle (Fig. S13), and helix α2 binds to the interface of two adjacent nozzle proteins of gp12 and the turn of the antiparallel α-helices of gp14 (Fig. S11D and S13). The gp14–gp6.7 complex embedded in the open nozzle extends the tail channel, with the distal end of this channel connecting to LPS (Fig. 1D, 3A and B).

## DISCUSSION

**Fig 4:**
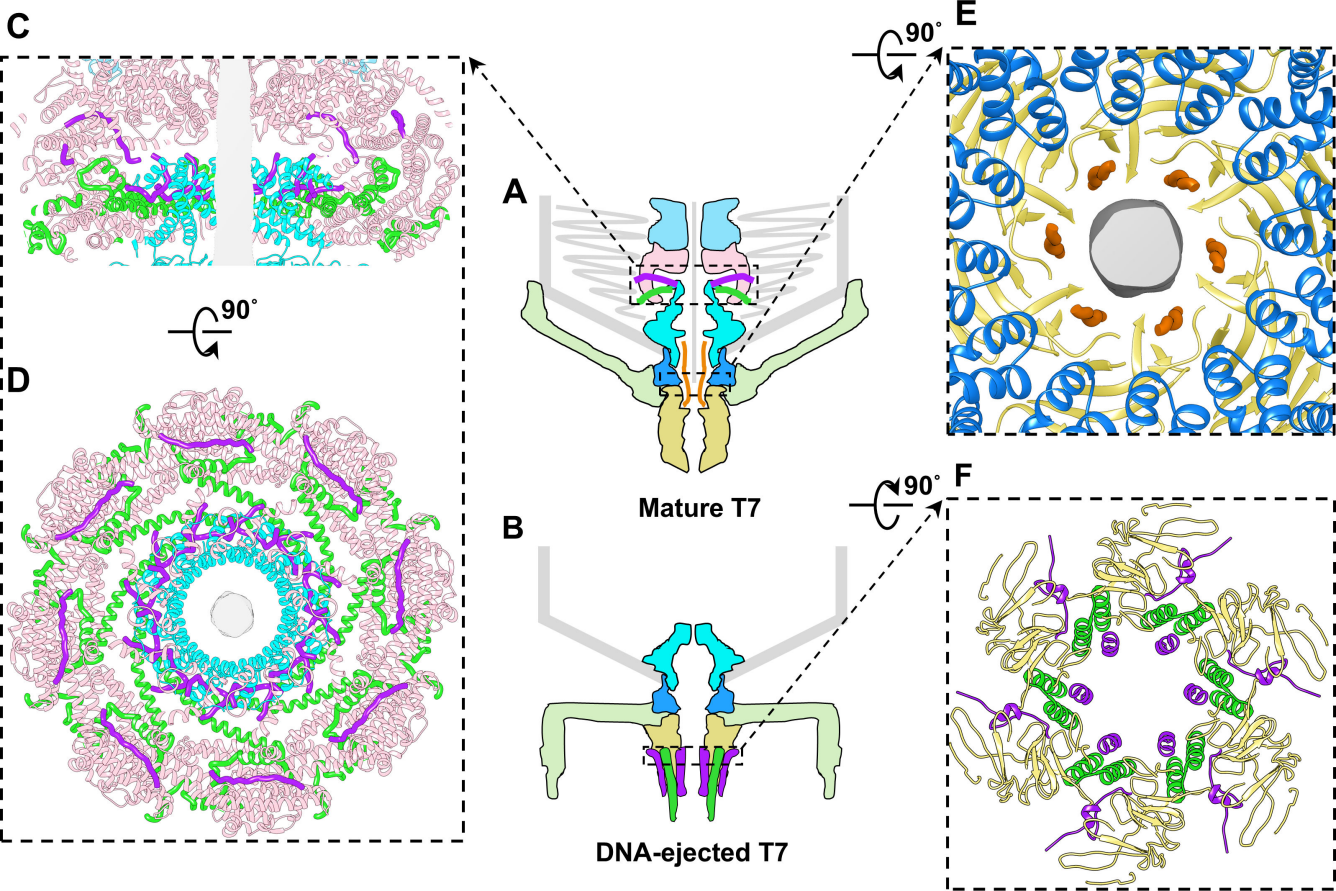
Conformational and location changes from mature to DNA-ejected states of three small proteins of gp14, gp6.7, and gp7.3. (**A**) Diagram of mature T7. (**B**) Diagram of DNA-ejected T7. (**C, D**) Slab (**C**) and cross-section (**D**) views of columns in A showing interactions among proteins gp6.7, gp14, and gp15, and portal. (**E**) Cross-section views of columns in A showing interactions among gp7.3, adaptor, and nozzle. (**F**) Cross-section views of columns in B showing interactions between gp6.7, gp14, and nozzle. Color coding is identical to that in Fig. 1.

During infection, the tail and core proteins of podophages undergo a series of conformational changes for the effective ejection of the genome into the cytoplasm of the host cell. In this study, we applied defocus refinement with Z-height correction to resolve the *in situ* structures of the core, portal, and tail, overcoming previous challenges for determining small proteins within large phage particles. We resolved the structures of the proteins involved in dsDNA delivery in both mature and DNA-ejected T7 at resolutions of approximately 3 Å. On the basis of the high-resolution density maps, we built atomic models for the previously unidentified small proteins of gp14, gp6.7, and gp7.3 in mature T7 as well as gp14 and gp6.7 in DNA-ejected T7. By using structures of these proteins in the aforementioned two states, the mechanism for core protein and DNA ejection can be illustrated. The three small proteins of gp14, gp6.7, and gp7.3, together with gp15 and gp16, undergo conformational changes from the mature to DNA-ejected states (Fig. 4A and B).

In the mature T7, gp15 octamer and gp16 tetramer form the major body of the core. In addition, twelve copies of gp6.7 and eight copies of gp14 serve to bridge the helical barrel domain of the portal and the octameric gp15, thereby stabilizing the core on the portal (Fig. 4C and D). Our structural result for gp6.7 is consistent with the biochemical result, suggesting that gp6.7 loss in the T7 infection process causes the disaggregation and ejection of core proteins (27).

A previous biochemical result demonstrated that gp7.3 is essential for T7 assembly, resulting in forming an infective phage, but it is not required in the mature virion (27). Our structure revealed that six copies of gp7.3 are located at the junction of the adaptor and nozzle within the tail channel (Fig. 1B and 4E), suggesting that gp7.3 might serve as a structural scaffold to assist the assembly of the nozzle into the adaptor. In addition, the biochemical results also showed that T7 particles assembled in the absence of gp7.3 contain tail fibers but fail to adsorb to the outer membrane of cells (27). Considering that the nozzle plays an important role in the T7 adsorption (16), a stable nozzle assembly in the tail, which is assisted by the gp7.3 scaffold, might be required during the T7 adsorption.

In the DNA-ejected T7, six copies of gp14 and twelve copies of gp6.7 are ejected from the capsid and cooperate again, forming an extended tail channel in the bacterial outer membrane (Fig. 4F). A previous study showed that T7 carrying a mutant gp6.7 adsorbed normally, but exhibited lower ejection efficiency than wild-type T7 (27) suggesting that the extended gp14 channel on the bacterial outer membrane is further reinforced by the core protein gp6.7. The locations of gp14 and gp6.7 in DNA-ejected T7 suggest that the ejection of gp14 and gp6.7 onto the outer membrane occurs in preparation for the ejection of gp15 and gp16. According to our structures, gp6.7 is closely involved in the assembly of the core complex within mature T7 and the formation of a trans-envelope channel during T7 infection. Therefore, we suggest that gp6.7 is the fourth core protein in addition to gp14, gp15, and gp16.

Notably, eight copies of gp14 participate in the formation of the core in the mature virions, and only six copies of gp14 are involved in the formation of the extended channel of the DNA-ejected particles. We speculate that the eight copies of gp14 are to adapt the symmetry–mismatch between the twelve-fold ring of gp6.7 loop I around the portal and eightfold ring of gp15 during the core assembly. The eight copies of gp14 could ensure a stable assembly of the octameric gp15 ring on the portal. During infection, only six copies of gp14 interact with the nozzle and participate in the formation of the extended channel because the nozzle is formed by six copies of gp12 and may provide only six binding sites for gp14. In addition, the reduction of the protein copy number from the mature state to the DNA-ejected state might accommodate the appropriate size of the extended channel for DNA delivery. Whether the two missing gp14 molecules have any biological function or are simply released into the cell envelop or cytoplasm remains to be elucidated.

Cryo-electron tomography analysis of the T7 at successive stages of infection revealed that after DNA is ejected into the host cytoplasm, the extended channel formed by the core proteins is observed to collapse or disassemble in host cell, possibly to avoid the long-term existence of the trans-envelope channel that affect cell integrity (16). This structural result is consistent with the biochemical result that both gp6.7 and gp7.3 are normally degraded by one or more cellular proteases (28, 29). Therefore, our structure of the extended channel in the LPS-induced DNA-ejected T7 could represent the extended channel before degradation (Fig. 4B and F). On the basis of our two-state T7 structures and previous biochemical study of gp6.7 and gp7.3 (27) and the biochemical and structural studies of core proteins gp14, gp15, and gp16 (23, 30, 31), we propose a detailed molecular mechanism for the ejection of T7 core proteins and DNA during infection (Fig. S14). First, T7 fibers randomly explore the cell receptor during initial infection (Fig. S14A and B). After the adsorption of all fibers onto the cell membrane, the tail axis becomes perpendicular to the cell, possibly triggering conformational changes of the tail nozzle, thereby opening the tail channel and ejecting the gp7.3 molecules (Fig. S14C). Subsequently, an ejection signal is transmitted to gp6.7 molecules, resulting in the ejection of the core proteins of gp14 and gp6.7 and the formation of an extended tail channel spanning the outer membrane of the host cell (Fig. S14D). Accompanied by the gp14 and gp6.7 ejection, the helical barrel domain of the portal, the octamer of gp15, and the tetramer of gp16 collapse, gp15 and gp16 are then ejected into the periplasm through the portal–tail channel due to the DNA pressure inside the capsid (Fig. S14E). The ejected core proteins of gp15 and gp16 form a hexameric elongated channel, which connects with the gp14–gp6.7 channel to span the periplasm and the inner membrane, and reaches the cytoplasm for DNA delivery (30, 34–37) (Fig. S14F).

## MATERIALS AND METHODS

### Preparation of mature T7 sample and LPS incubation assay

Mature and DNA-ejected T7 were prepared using an established method (23, 24, 29). In brief, the T7 phage was propagated before precipitation with 1 M NaCl and 10% polyethylene glycol 8000 and purified by ultracentrifugation (197,000×*g*, 12 h, 10°C) on 1.56 g/mL and 1.26 g/mL CsCl cushions. Two phage bands were separated after centrifugation. The upper and the lower bands were empty and mature particles observed by negative stain electron microscope, respectively. The lower band was collected and repeatedly ultracentrifuged on the aforementioned 1.56 g/mL CsCl cushions. The mature T7 particle band was next dialyzed overnight in phage buffer (50 mM Tris-HCl, 10 mM MgCl_2_, and 50 mM NaCl, pH 7.4) and collected for LPS incubation. Subsequently, the purified mature T7 particles were interacted with rough LPS (250 µg/mL) from *E. coli* serotype EH100 (Ra; Hycult Biotech, HC4046) in a water incubator at 37°C for 3 h. The T7–LPS mixture was then placed in an ice–water bath to stop the incubation; thus, the DNA-ejected T7 complexed with LPS was obtained.

### Data acquisition and image processing

The mature and DNA-ejected T7 particles complexed with the LPS were imaged using a Titan Krios G3i microscope equipped with a Gatan imaging filter and a K3 summit direct electron detector at 64,000× magnification, corresponding to a calibration size of 1.06 Å/pixel. The full dose for each movie was approximately ~35 e^-^/Å^2^. In total, 2,221 and 4,401 movies were recorded for mature and DNA-ejected T7 particles, respectively. The astigmatism and defocus values of each image were determined using the GCTF (38) package in RELION-3.1.4 (39). All viral particles were then automatically boxed using ETHAN software (40), and 75,594 and 23,461 particle images were extracted from the raw images of mature and DNA-ejected T7, respectively.

The icosahedral and symmetry–mismatch reconstructions of the mature and DNA-ejected T7 complexed with LPS were performed using previously described programs (23, 24). In brief, the icosahedral reconstruction of mature T7 was performed using our programs (41, 42) on the basis of the common-line algorithm (43, 44). The asymmetric structure of the entire mature T7 particle was then reconstructed at a resolution of 6.1 Å by using the symmetry–mismatch reconstruction method (32). In brief: (i) for each two-dimensional particle image, the correlation coefficients between 60 equivalent icosahedral projection images and the particle image were calculated to obtain the best matching asymmetric orientation; (ii) using the new orientation, the asymmetric structure was reconstructed without the imposition of any symmetry; (iii) using the asymmetric structure from step (ii) as the initial model, steps (i) and (ii) were repeated until the resolution of the asymmetric structure could no longer be improved.

Using the local refinement and reconstruction method (23), we refined the orientations and center parameters of the core–portal–tail complex in mature T7 to a resolution of 3.9 Å by imposing two-fold symmetry. Next, we reconstructed the core complex and portal–tail complex independently in mature T7 until most of the orientations did not change. Using our latest programs, we further iteratively refined the Z-heights, orientations, and center parameters of the core complex, portal, and portal–tail complex in mature T7, improving the structural resolutions to 3, 2.5, and 2.7 Å by imposing, four-, twelve-, and six-fold symmetry, respectively. In the same image processing protocol, we resolved the entire DNA-ejected T7 structure at a resolution of 8.2 Å without the imposition of symmetry, as well as the structure of the portal–tail complex of DNA-ejected T7 at a resolution of 3.5 Å by imposing a six-fold symmetry.

### Atomic model building and refinement

Our previous atomic models of the portal protein gp8, adaptor protein gp11, nozzle protein gp12, and fiber protein gp17 in mature T7 and DNA-ejected T7, the core proteins gp15 and gp16 in mature T7, and core protein gp14 in DNA-ejected T7 were used to further refined based on our new density maps (23). Moreover, the models of the core protein gp6.7 and gp14, tail protein gp7.3 in mature T7, and gp6.7 in DNA-ejected T7 were manually built on the basis of our cryo-EM density map by using the COOT software (45). All models were refined using real-space refinement implemented in Phenix (46). Refinement and validation statistics are presented (Table S1).

## Data Availability

The electron density maps and atomic coordinates have been deposited in the Electron Microscopy Data Bank under accession codes EMD-61906, EMD-61907, EMD-61908, EMD-61909, EMD-61910, and EMD-61911 and in the Protein Data Bank under accession codes 9JYY, 9JYZ, and 9JZ0.

## SUPPLEMENTAL MATERIAL

Supplemental material jvi.01249-24-s0001.pdf Figures S1 to S14 and Table S1.

ASM does not own the copyrights to Supplemental Material that may be linked to, or accessed through, an article. The authors have granted ASM a non-exclusive, world-wide license to publish the Supplemental Material files. Please contact the corresponding author directly for reuse.
