# Supplementary material for: Conformational changes in and translocation of small proteins: insights into the ejection mechanism of podophages
Source: J Virol. 2024 Dec 20;99(1):e01249-24. doi: 10.1128/jvi.01249-24 (PMC11784390; doi:10.1128/jvi.01249-24)
Supplement: Supplemental material — Figures S1 to S14 and Table S1. [file jvi.01249-24-s0001.pdf]

Supplementary information for

Conformational changes in and translocation of small proteins: Insights into the ejection mechanism of podophages

Jing Zheng[1,#], Hao Xiao[1,2,#], Hao Pang[1,2], Li Wang[3], Jingdong Song[2], Wenyuan Chen[1,*], Lingpeng Cheng[1,*], and Hongrong Liu[1,*]

[1]Institute of Interdisciplinary Studies, Key Laboratory for Matter Microstructure and Function of Hunan Province, Key Laboratory of Low-dimensional Quantum Structures and Quantum Control, School of Physics and Electronics, Hunan Normal University, Changsha 410082, China.

[2]State Key Laboratory of Infectious Disease Prevention and Control, National Institute for Viral Disease Control and Prevention, Chinese Center for Disease Control and Prevention, Beijing 100052, China.

[3]Department of Microbiology, College of Life Science, Hunan Normal University, Changsha 410081, China.

[#]These authors contributed equally to this work.

*Correspondence to: wenyuanchen@hunnu.edu.cn, lingpengcheng@hunnu.edu.cn, and hrliu@hunnu.edu.cn

Competing interests: The authors declare no conflict of interest.

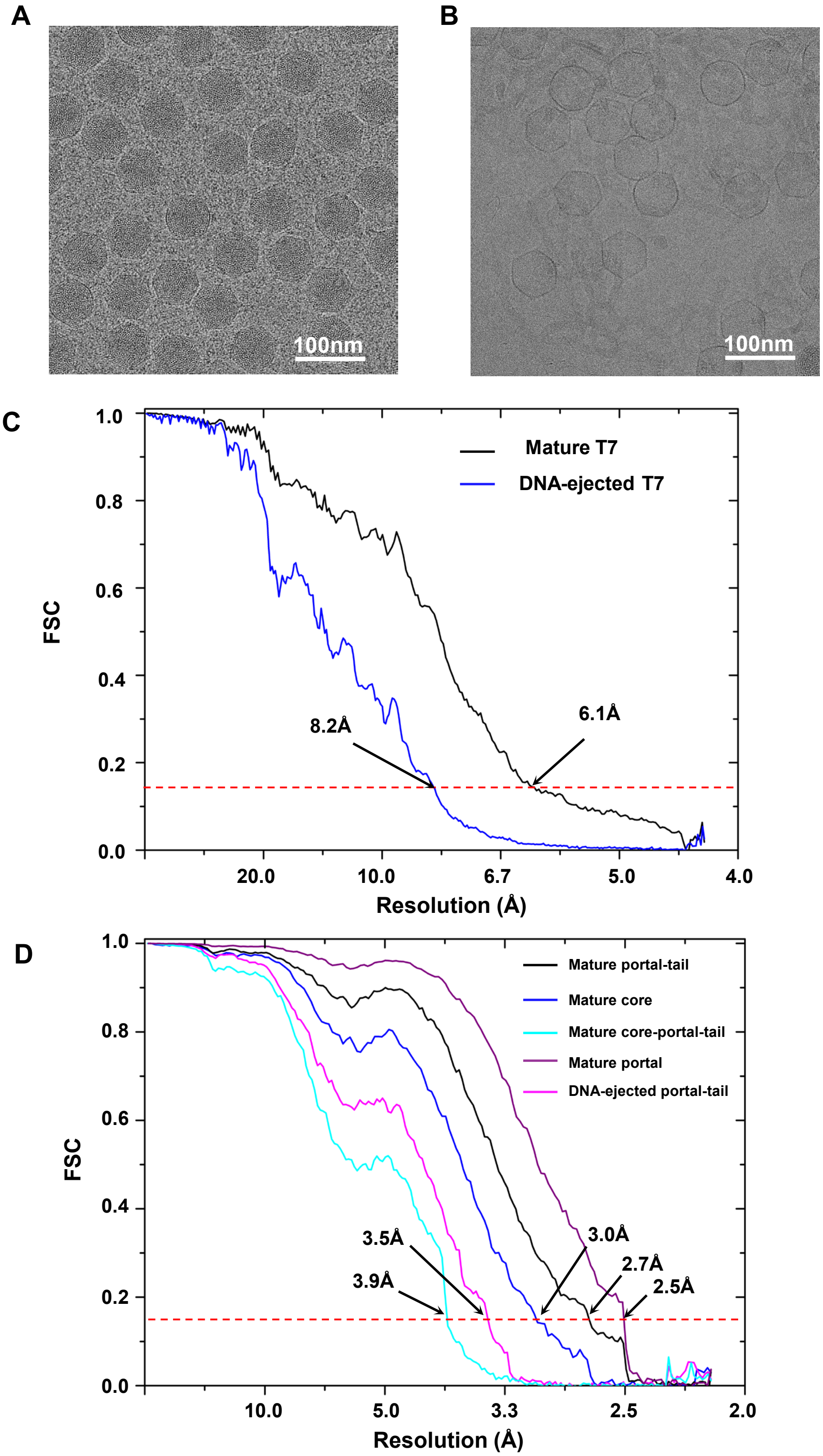

Fig. S1 Cryo-EM images and reconstruction resolutions. A) and B) Representative cryo-EM images of mature (A) and DNA-ejected (B) T7. **C)** Resolutions of asymmetric reconstructions of entire mature T7 (black line) and entire DNA-ejected T7 in complex with LPS (blue line). **D)** Resolutions of local reconstructions of portal-tail complex (black line), core complex (blue line), core-portal-tail complex (cyan line), and portal (purple line) in mature T7, as well as those of portal-tail complex (magenta line) in DNA-ejected T7.

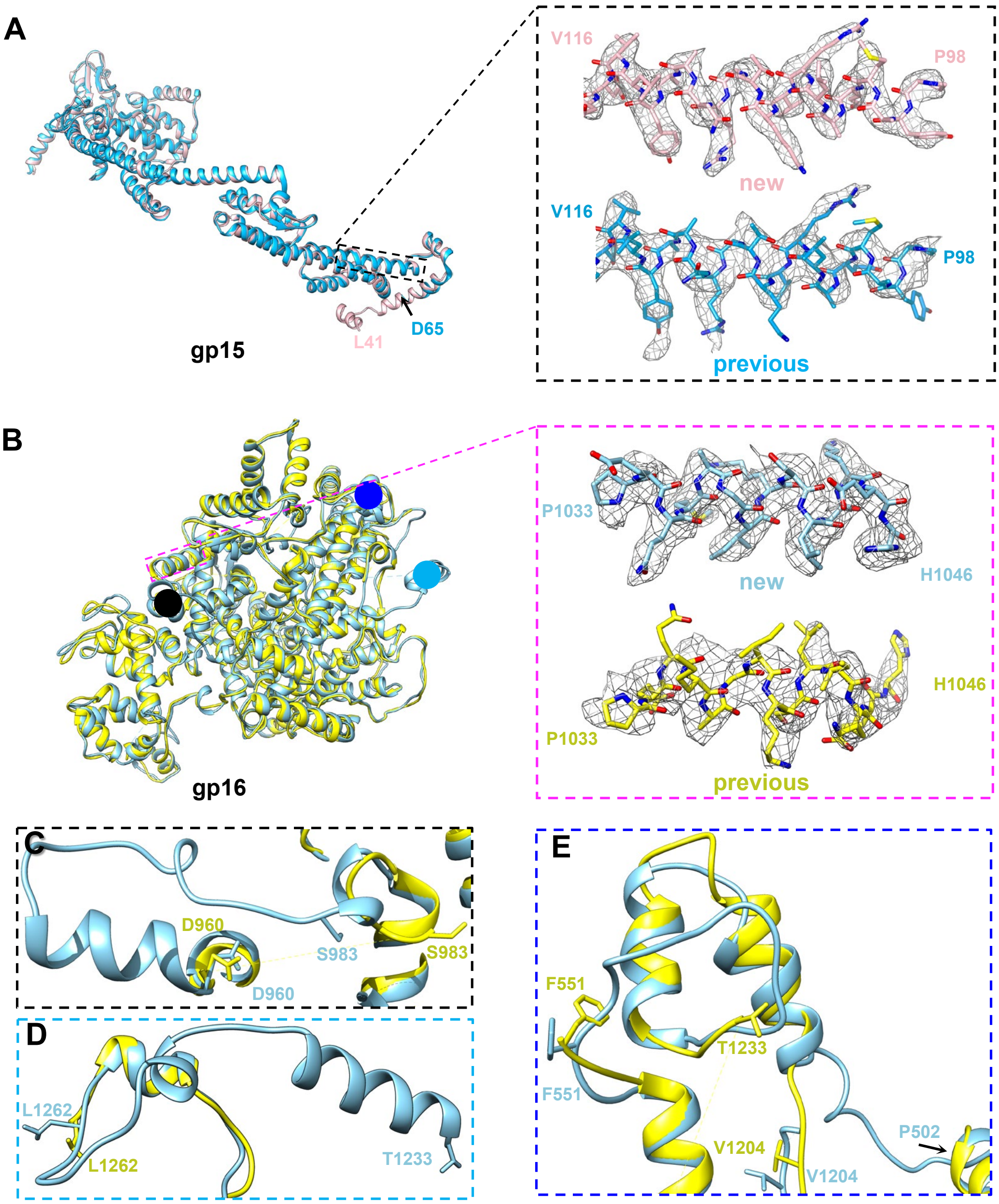

Fig. S2 Comparison of new and previous atomic models of gp15 and gp16. A) Superposition of atomic models (left) and density difference (right) of new (pink) and previous (deep sky blue, PDB ID: 7EYB) atomic models of gp15. **B-E)** Superposition of atomic models (left) and density difference (right) of the new (sky blue) and previous (yellow, PDB ID: 7EYB) atomic models of gp16 (B). Zoomed-in views showing newly resolved segments of gp16, residues D960 to S983 (C), T1233 to L1262 (D), and P502 to F551 (E).

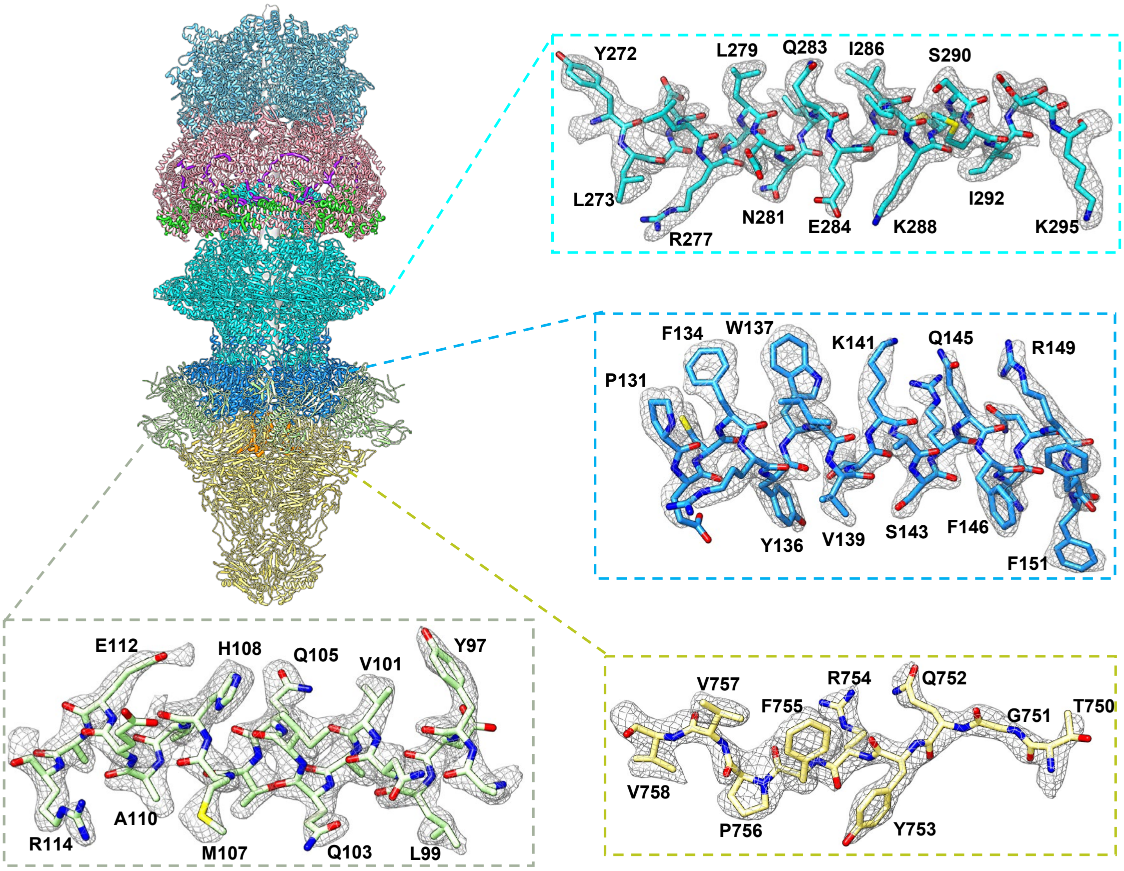

Fig. S3 Ribbon models of core-portal-tail complex in mature T7, and zoomed-in views of density maps (mesh) superimposed on their atomic models (sticks). Color coding is identical to that in Fig. 1B.

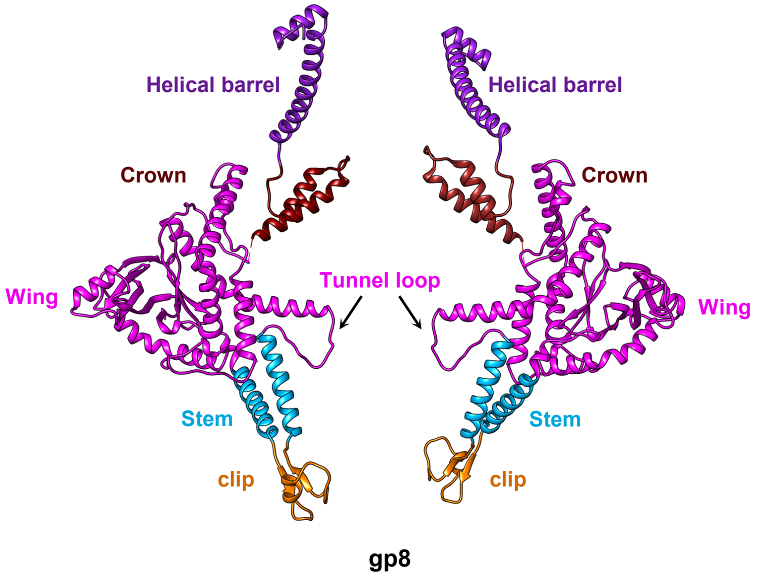

Fig. S4 Atomic models of portal gp8 in mature T7.

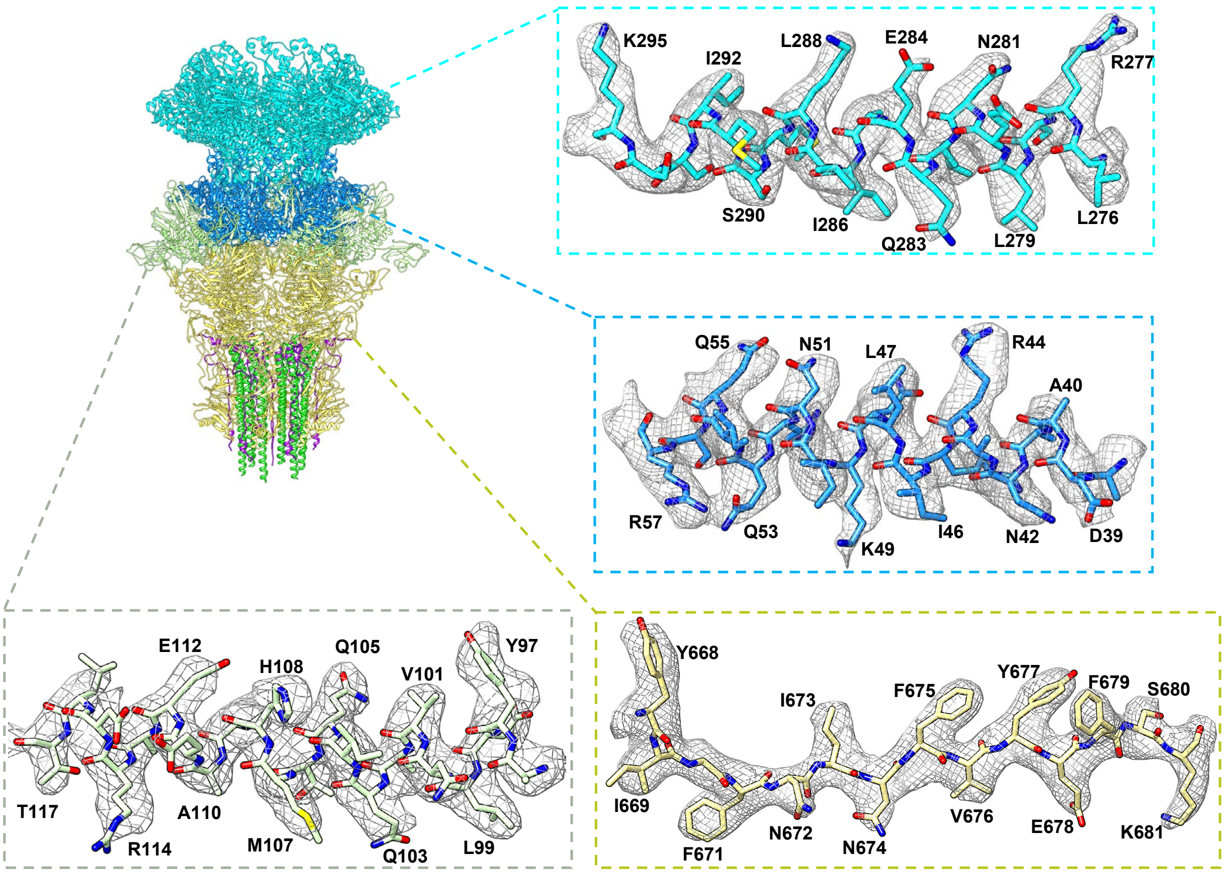

Fig. S5 Ribbon models of portal-tail complex in DNA-ejected T7, and zoomed-in views of density maps (mesh) superimposed on their atomic models (sticks). Color coding is identical to that in Fig. 1E.

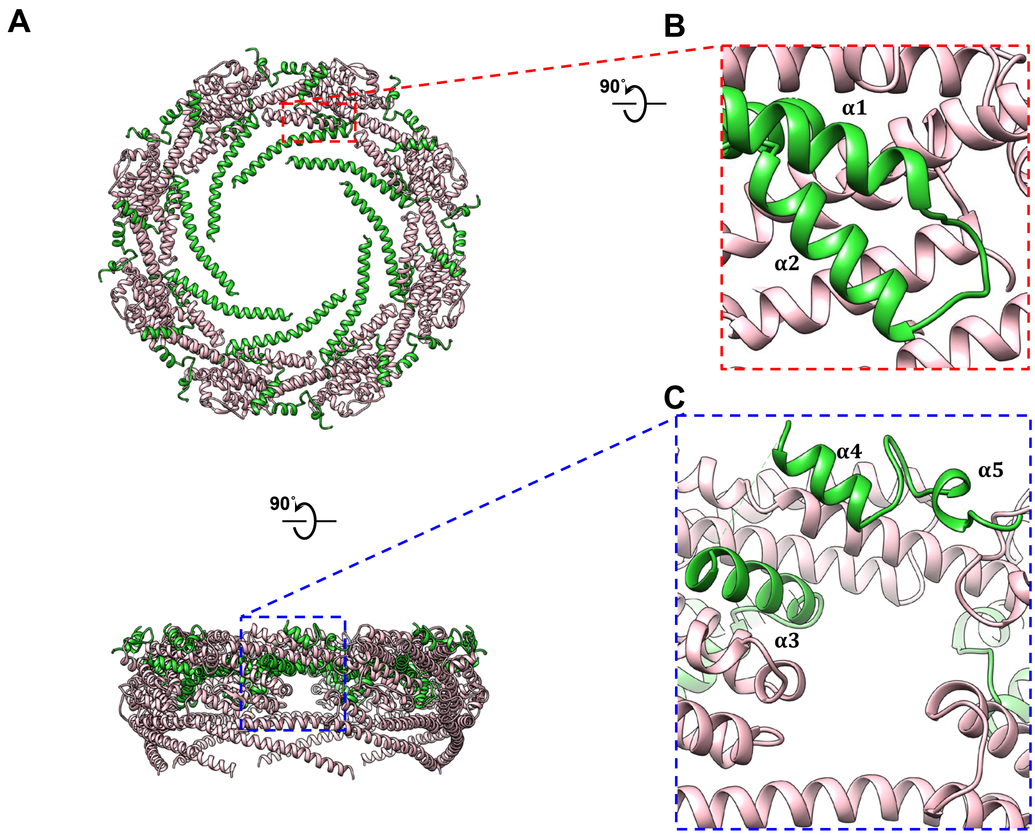

Fig. S6 Interactions between core proteins gp14 and gp15 in mature T7. A) Bottom and side views of interactions between core proteins gp14 (lime green) and gp15 (pink). **B)** Zoomed-in view of interactions among gp15 and α1 and α2 in gp14. **C)** Zoomed-in view of interactions among gp15 and α3, α4, and α5 in gp14 .

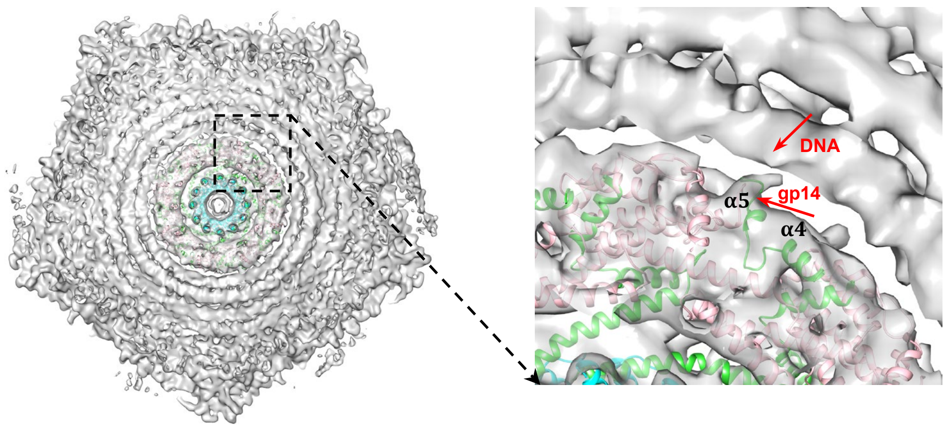

Fig. S7 C-terminal domain (residues 130-196) in gp14 (lime green) close to T7 DNA (gray).

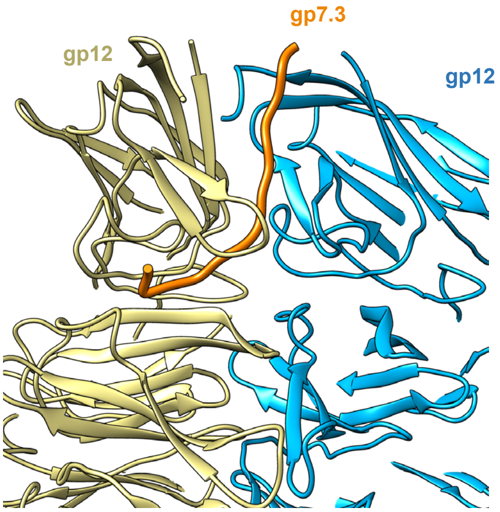

Mature T7

Fig. S8 Interactions between 2 nozzle proteins gp12 and small protein gp7.3 in mature T7.

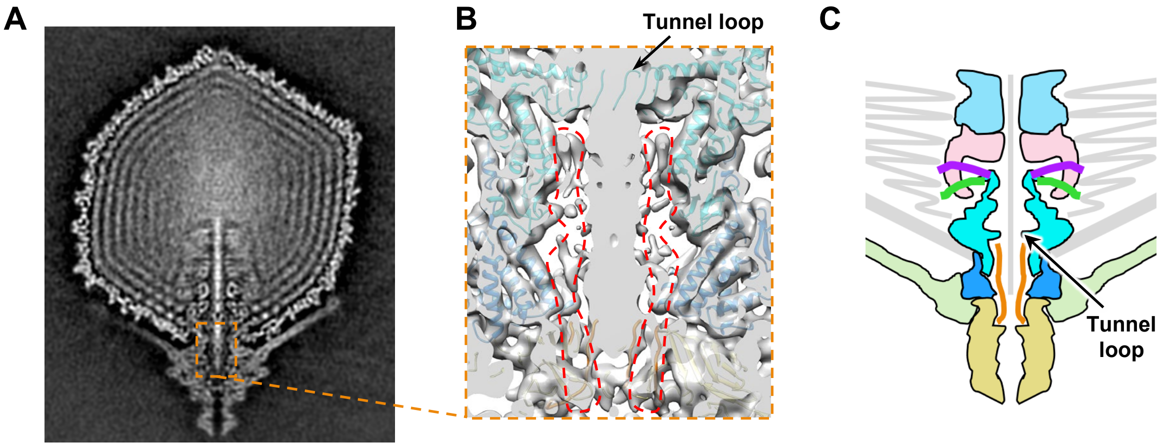

Fig. S9 Distribution of small protein gp7.3 in mature T7. A) Central slice view of asymmetric structure of mature T7. **B)** Zoomed-in views of atomic models superimposed on medium resolution density map (transparent gray), showing possible density maps of gp7.3 designated using red dashed lines. **C)** Diagram of mature T7 showing possible location of gp7.3 (orange). Color coding is identical to that in Fig. 1A.

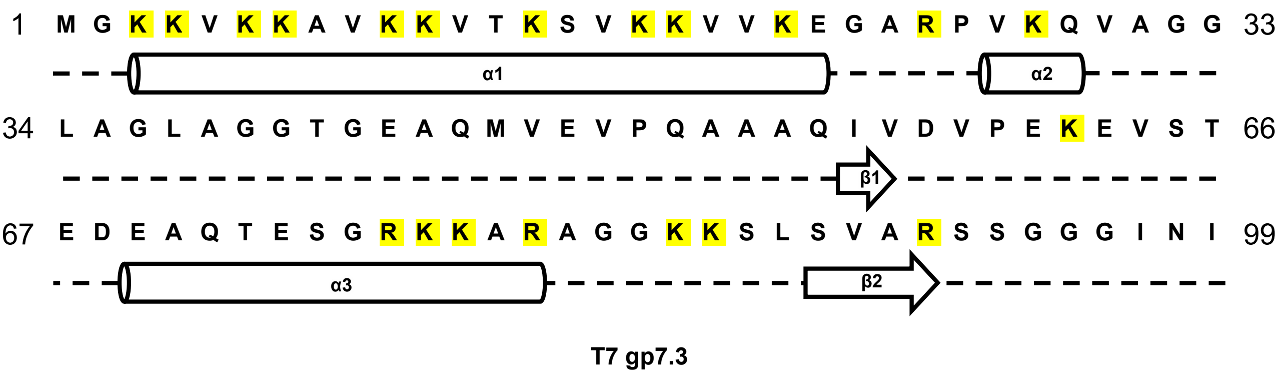

Fig. S10 Amino acid sequence and secondary structure elements of gp7.3 in phage T7. All basic residues are labeled in yellow.

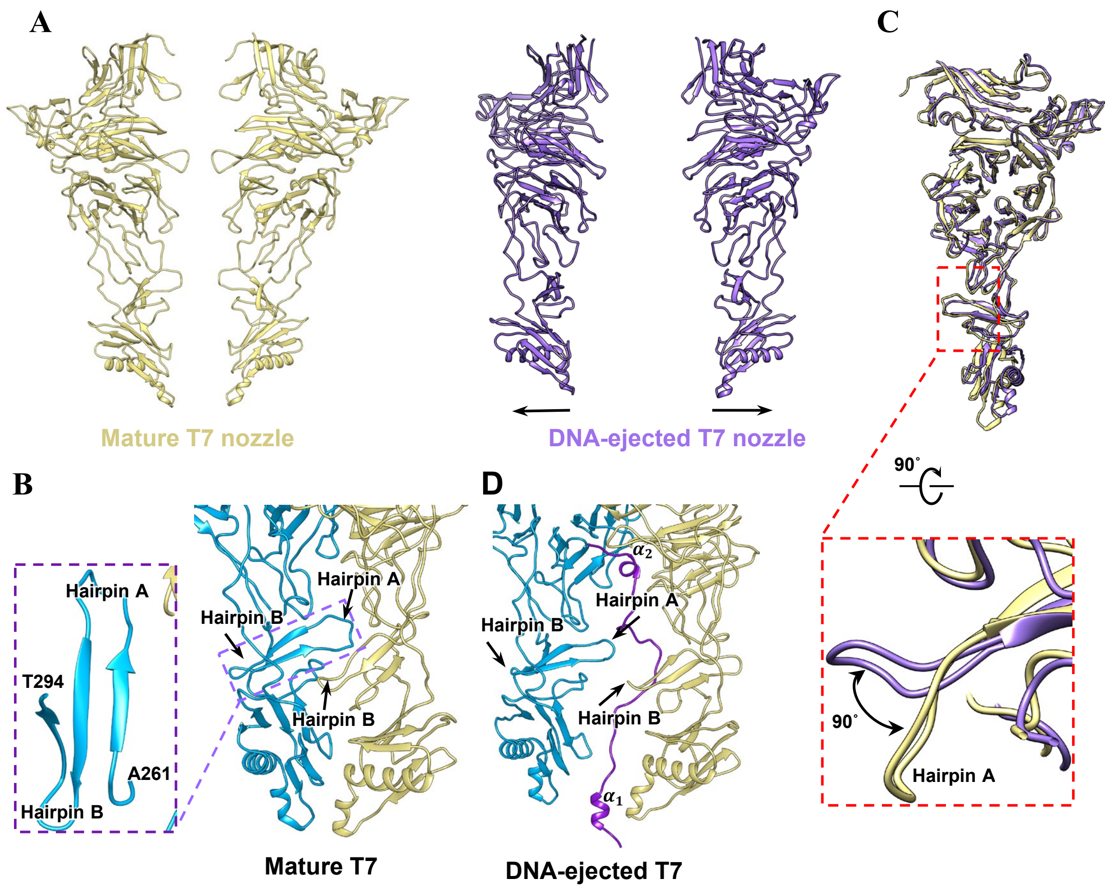

Fig. S11 Conformational changes of nozzle gp12 from mature T7 to DNA-ejected T7 states. A) Slab views of atomic models of nozzle in mature T7 and DNA-ejected T7. **B, D)** Distance between hairpins A and B from 2 adjacent gp12 proteins in mature T7 (B) and DNA-ejected T7 (D). Conformer B α2 of gp6.7 (purple) binds to interface of 2 adjacent gp12 proteins. **C)** Superposition of atomic models of gp12 in mature (khaki) and DNA-ejected (medium purple) T7.

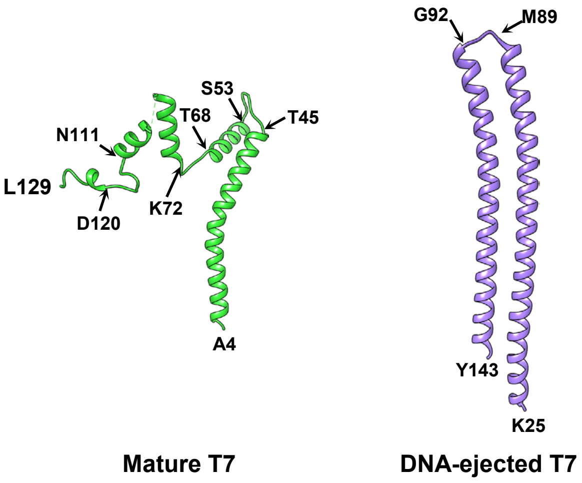

Fig. S12 Structural comparison of atomic models of gp14 in mature and DNA-ejected T7.

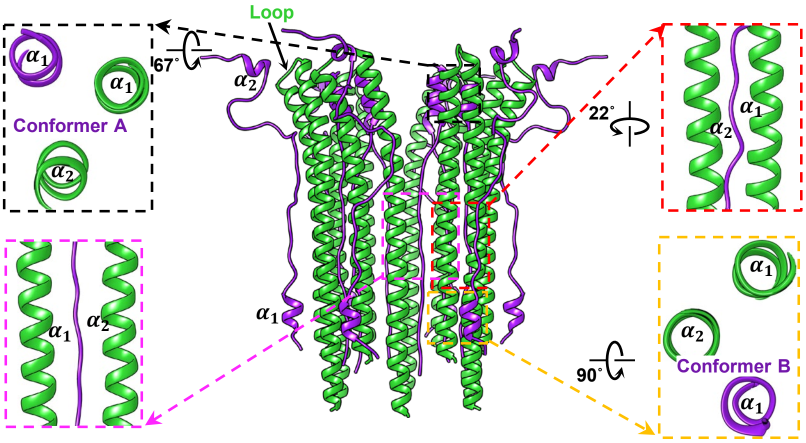

Fig. S13 Interactions between gp14 and gp6.7 in DNA-ejected T7. In left insets, conformer A of gp6.7 interacts with gp14. In right insets, conformer B of gp6.7 interacts with gp14. Color coding is identical to that in Fig. 1E.

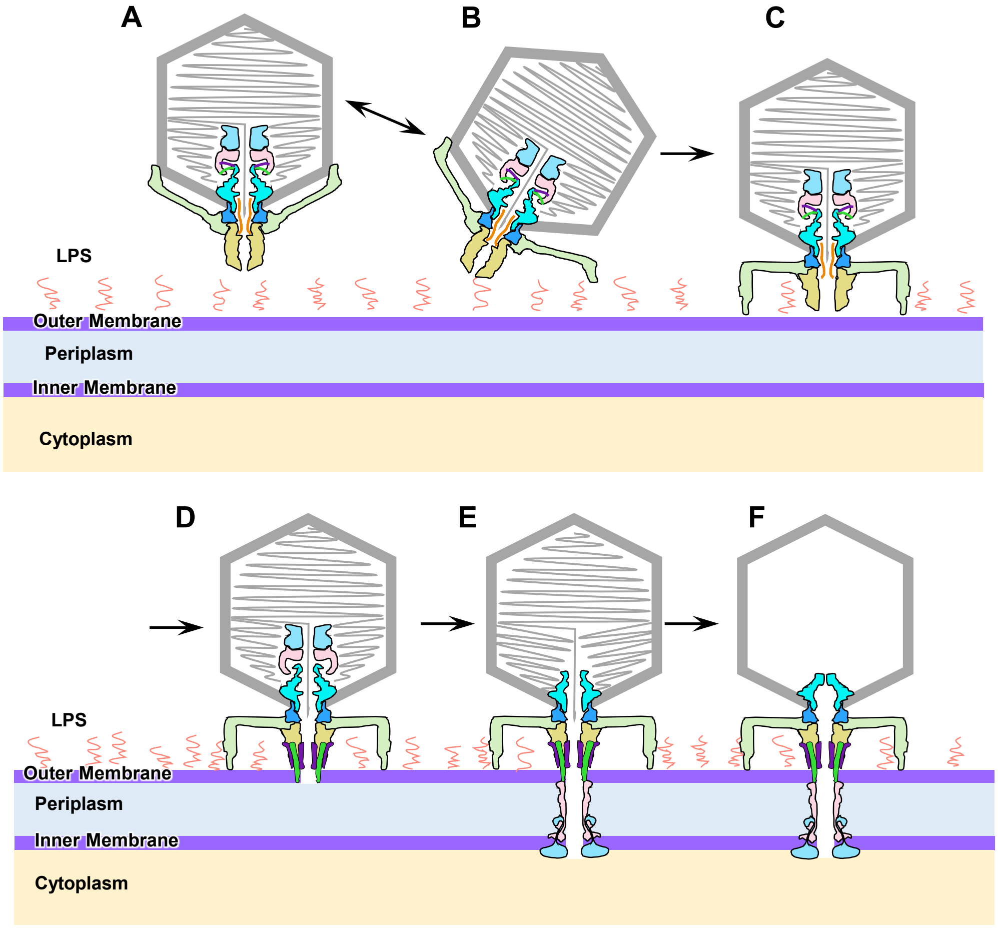

Fig. S14 Schematic of T7 infection and DNA-ejection pathway. A) Mature T7. **B**) Fibers randomly explore cell surface to find receptors. **C**) T7 reorients for binding to cell surface, and triggering opening of tail channel and gp7.3 ejection. **D)** Proteins gp6.7 and gp14 exit to form an extended channel. **E)** Proteins gp15 and gp16 exit from head and form channel throughout periplasm and inner membrane into cytoplasm. **F)** DNA is ejected into the host cytoplasm. Color coding is identical to that in Fig. 1.

Supplementary Tables

Table S1. Refinement and model statistics of T7.

Data collection		
Electron microscopy	Titan Krios G3i	
Pixel size((Å))	1.06	
Defocus range	0.5 to 4.0um	
Total movie-mode micrographs	mature phage T7	DNA-ejected T7
	2,221	4,401

Symmetry-mismatch reconstruction		
	mature phage T7	DNA-ejected T7
Total particles	75,594	23,461
Resolution(Å)	6.1	8.2
EMDB ID	EMD-61906	EMD-61907

Local reconstruction				
	mature phage T7			DNA-ejected T7
	core	portal-tail	core-portal-tail	portal-tail
Total particles	45731	65620	45731	23461
Resolution(Å)	3.0	2.7	3.9	3.5
B-factors	90	70	120	100
EMDB ID	EMD-61909	EMD-61910	EMD-61908	EMD-61911

Atomic models refinement/statistics (phenix.real_space_refine）			
	mature phage T7		DNA-ejected T7
Protein	core	portal-tail	portal-tail
PDB ID	9JYY	9JYZ	9JZ0
Model Resolution in Refinement(Å)	3.5	3.2	3.5
Total Residues	11196	15984	15342
CC (model to map fit)	0.7842	0.7607	0.8025
Ramachandran most favorable (%)	93.94	97.90	94.35
Ramachandran additionally allowed (%)	5.62	2.03	5.31
Ramachandran disallowed (%)	0.45	0.08	0.34
